# Small Intestinal Amyloidosis: A Rare Cause of Diverticular Disease

**DOI:** 10.1155/2014/362835

**Published:** 2014-06-05

**Authors:** Gabriel M. Groisman, Hector I. Cohen

**Affiliations:** ^1^Institute of Pathology, Hillel Yaffe Medical Center, 38100 Hadera, Israel; ^2^Institute of Pathology, Western Galilee Hospital, 22100 Naharyia, Israel

## Abstract

Systemic amyloidosis frequently involves the small intestine. However, its association with diverticular disease has been seldom reported to date. To draw attention to this rare but potentially harmful association, we herein present an additional case of small bowel diverticular disease associated with amyloidosis.

## 1. Introduction

Diverticular disease is seldom observed in the small bowel [1]. Its actual frequency is hard to estimate as in most cases it is found incidentally and is often not documented. However, the incidence reported in enteroclysis studies of the small bowel is 2–2.3%, comparable to autopsy data presenting an incidence of 1.3–4.6% [2–4].

Jejunoileal diverticula result from mucosal and submucosal herniation through the muscular layer of the bowel wall in places of minor resistance to the intraluminal pressure. In most cases the etiology is unclear. The pathogenesis is probably related to abnormalities of the muscular layers or the myenteric plexus that cause intestinal dyskinesia resulting in intestinal contractions which generate increased intraluminal pressure, favoring diverticula formation through the weakest point of the bowel [5, 6].

Amyloidosis (derived from immunoglobulin light chain [AL, formerly termed “primary”] or derived from amyloid serum A protein [SAA, formerly termed “secondary”]) commonly affects the small intestine and it is often associated with dysmotility disorders or dyskinesia of the small and large bowel. However, small bowel amyloidosis is only very rarely associated with small bowel diverticular formation with only two cases described so far [7, 8]. In order to draw attention to this rare association, we herein present an additional uncommon case of small bowel diverticular disease associated with amyloidosis.

## 2. Case Presentation

A 91-year-old man with a medical history of multiple myeloma and respiratory insufficiency was admitted with a 24-hour history of nausea and colicky abdominal pain located in the epigastrium. He also reported constipation for the past 3 days with only one bowel movement and a recent episode of enterorrhagia. On admission, clinical examination revealed generalized mild abdominal tenderness, with local guarding to the left abdomen and rebound tenderness. Mild bowel sounds were present with high frequency. Rectal examination revealed an empty rectum. The blood count revealed leukocytosis (18,500/mm^3^) with left shift (90% polymorphonuclears) and elevated C-reactive protein of 11.1 mg/dL. After 3 hours, the patient became febrile, tachycardic (110 beats per minute), and hypotensive (80/50 mmHg) with diffuse abdominal tenderness, rebound, and guarding. An ultrasound examination of the abdomen revealed dilated small bowel loops with scattered diverticula and free fluid in the abdominal cavity. He underwent an emergency laparotomy at which 197 cm of ischemic small bowel was identified and excised. Despite postoperative therapy in the intensive care unit, the patient developed severe coagulopathy and acidosis and finally died 3 days following surgery. Permission for autopsy was not granted.

## 3. Pathologic Examination

Gross examination showed a segment, 197 cm in length, of small intestine with a grey, smooth, and focally hemorrhagic serosal surface. The mucosa was dark and purple as a result of extensive hemorrhages and revealed superficial ulceration. Sections of the bowel wall displayed numerous diverticular structures.

**Figure 1:**
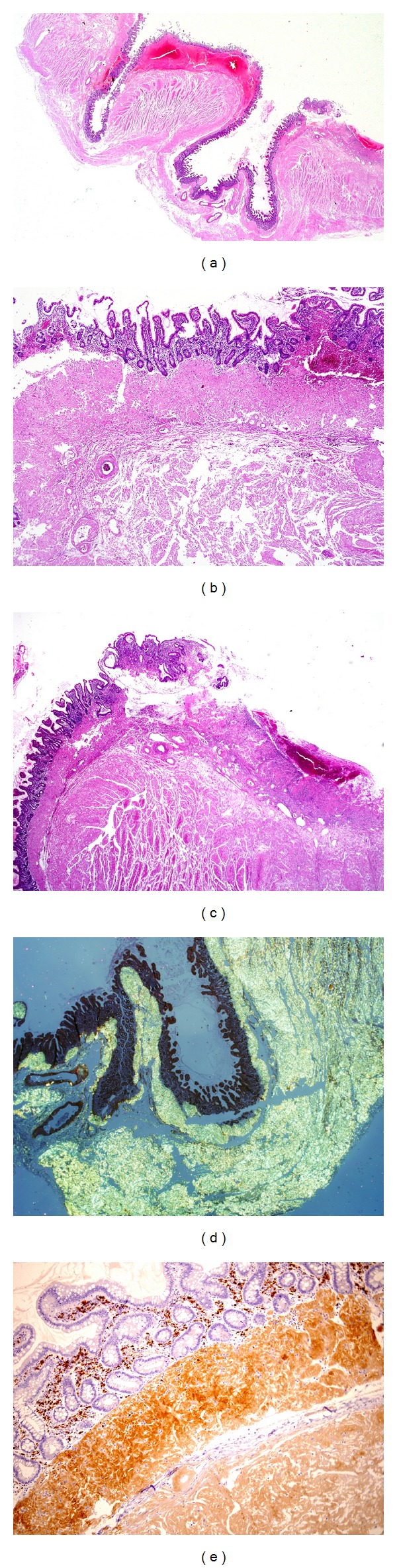
(a) Whole mount section of the small intestine showing diverticular disease (hematoxylin and eosin stained section, magnification ×8). (b) and (c) show extensive deposition of eosinophilic amorphous material (amyloid) in the muscularis mucosa, submucosa, and muscularis propria layers of the small bowel. Ischemic mucosal ulceration and hemorrhages are present in (c) (hematoxylin and eosin stained sections, (b) magnification ×40 and (c) magnification ×20). (d) Congo red stain under polarized light shows extensive green birefringence, diagnostic of amyloid deposition (magnification ×2). (e) Immunostain for kappa light chain shows extensive AL amyloid deposition within the muscularis mucosa, muscularis propria, and blood vessel walls (magnification ×10).

Histologic sections of the small intestinal wall displayed ischemia-related changes, mainly mucosal, numerous diverticula and deposits of a hyaline, eosinophilic, and homogeneous material on blood vessel walls and in the muscularis propria, submucosa and muscularis mucosa layers (Figures 1(a), 1(b) and 1(c)). These deposits proved to be AL type amyloidosis as they displayed orange to green birefringence under cross-polarized light with the Congo red staining (Figure 1(d)), and reacted to immunohistochemical stain for amyloid P-component and for kappa light chain (Figure 1(e)) while lambda light chain was negative.

## 4. Discussion

Amyloidosis is characterized by the extracellular deposition of diverse types of amyloid proteins. These proteins have a typical fibrillary appearance under electron microscopy. Their arrangement in a beta-pleated sheet formation provides the binding sites for the Congo red dye used for the diagnosis as it conveys typical apple-green birefringence when examined under polarized light. Yet, despite the morphological and histological uniformity of the amyloid protein, it is clear that several biochemical types of the amyloid protein exist. The most common form of amyloidosis is amyloidosis AL (amyloidosis derived from monoclonal immunoglobulin light chain, formerly termed “primary”), which is associated with plasma cell dyscrasias, including multiple myeloma. The second most common form is systemic (AA) amyloidosis, caused by deposition of fragments of the acute phase reactant, serum amyloid A protein. This type is associated with chronic inflammatory diseases and infections, including tuberculosis, familial Mediterranean fever, rheumatoid arthritis, and Crohn's disease [9, 10].

Gastrointestinal (GI) involvement is common in all types of amyloidosis (primary and secondary), ranging from 85% to 100% [11–13], with the small intestine most commonly affected [14]. Amyloid deposition is seen in the mucosa, the submucosal connective tissue, the muscularis mucosa, and muscularis propria, within nerves and mostly within blood vessel walls. Patchy involvement of the gastrointestinal tract is asymptomatic in most cases. With increased deposition, a variety of symptoms, such as bleeding, motility disorders, and malabsorption, may occur [15–17]. The deposition of amyloid within blood vessel walls may lead to bowel ischemia, mucosal atrophy and ulceration, and eventually perforation [18].

In our case, the extensive deposition of amyloid in the small intestine caused ischemic changes due to blood vessel stenosis. However, it also led to diverticula formation due to amyloid deposition in the muscular layers. Patel et al. [7] suggested that amyloid deposition in the enteric muscular layer, which leads to mural weakness and dysmotility, is the etiological factor that produces diverticula.

Even though amyloidosis is frequently seen in the small bowel, it represents an extremely rare cause of diverticular disease with only two cases reported to date [7, 8]. The first one, described in 1993, was a 72-year-old male who presented with peritonitis secondary to a perforated large jejunal diverticulum. A 10 cm long segment of jejunum was resected. Grossly, it showed numerous filiform polyps and three mushroom-shaped diverticula including a perforated one. Microscopic examination, including Congo red stain, revealed extensive deposition of amyloid (AL type presumed by electrophoresis) in the submucosa, muscularis mucosa, muscularis propria, and blood vessels. At the edge of the perforation, most of the muscularis propria had been replaced with amyloid. The patient had an uneventful postoperative course and upper gastrointestinal and small bowel series demonstrated multiple duodenal and jejunoileal diverticula. Duodenal biopsies were positive for amyloid [7]. The second case, reported in 2003, was a 91-year-old female who underwent a laparotomy for a small bowel obstruction. There were segmental transmural small bowel necrosis and a perforated area. Two segments of small bowel totaling about 80 cm were excised. They showed necrotic changes and multiple diverticula ranging from 2.0 to 3.0 cm in diameter. Two of the diverticula were perforated. Microscopically, most blood vessels displayed thickened walls and narrowed lumina. Congo red, lambda, kappa, and AA immunostain showed AL (lambda) amyloid deposition in blood vessels and submucosa. Postoperatively, the patient developed multiple medical problems and died 18 days following surgery [8]. Our patient is similar to the previously reported ones as he had ischemic changes and diverticular disease in the small bowel caused by amyloid deposition in blood vessel walls and in the muscular layer. In summary, all 3 cases occurred in elderly patients who presented with abdominal symptoms due to severe small bowel ischemia and diverticulosis caused by amyloidosis. Interestingly, in all these cases the amyloid type leading to vascular ischemia and diverticulosis was of AL type. This is most probably due to the fact that AL amyloidosis is the most common form of this disease. In fact, from the 1990s onwards, the AA/AL ratio was 1/17 to 1/38 due to fewer chronic infections and increasing recognition of AL amyloidosis [19, 20].

In conclusion, we have presented a rare case of diverticulosis and ischemia of the small bowel caused by massive AL amyloid deposition to draw attention to the possibility of these associations. It should be stressed that both ischemia and diverticular disease are serious events. They are of special concern because of their potential risk for infection, perforation, bleeding, and obstruction, all of which are severe complications that can compromise patient lives.
